# Comparison of Two Mice Strains, A/J and C57BL/6, in Caspase-1 Activity and IL-1*β* Secretion of Macrophage to *Mycobacterium leprae* Infection

**DOI:** 10.1155/2010/708713

**Published:** 2010-07-05

**Authors:** Tae Jin Kang, Geum Seon Lee, Se Kon Kim, Song Hou Jin, Gue Tae Chae

**Affiliations:** ^1^Institute of Chronic Disease, College of Pharmacy, Sahmyook University, 26-21 Kongnung 2-dong, Nowon-gu, Seoul 139-742, Republic of Korea; ^2^Institute of Hansen's Disease, College of Medicine, The Catholic University of Korea, Seoul 137-701, Republic of Korea

## Abstract

A/J mice were found to have amino acid differences in Naip5, one of the NOD-like receptors (NLRs) involved in the cytosolic recognition of pathogen-associated molecular patterns and one of the adaptor proteins for caspase-1 activation. This defect was associated with a susceptibility to *Legionella* infection, suggesting an important role for Naip5 in the immune response also to other intracellular pathogens, such as *Mycobacterium leprae*. In this study, the immune responses of macrophages from A/J mice against *M. leprae* were compared to those of macrophages from C57BL/6 mice. Infection with *M. leprae* induced high levels of TNF-*α* production and NF-*κ*B activation in A/J and C57BL/6 macrophages. Caspase-1 activation and IL-1*β* secretion were also induced in both macrophages. However, macrophages from A/J mice exhibited reduced caspase-1 activation and IL-1*β* secretion compared to C57BL/6 macrophages. These results suggest that NLR family proteins may have a role in the innate immune response to *M. leprae*.

## 1. Introduction

*Mycobacterium leprae* is an intracellular pathogen that often resides within specialized compartments and replicates in macrophages. This pathogen can induce macrophages to release inflammatory cytokines, such as IFN-*γ*, TNF-*α*, and IL-12, which are involved in the innate control of bacterial replication and the coordination of adaptive immune responses [1, 2].

 There are two major classes of pattern recognition receptor (PRR) in the innate immune system. Toll-like receptors (TLRs) detect conserved microbial components at the cell surface and within endosomes, whereas NOD-like receptors (NLRs) recognize a variety of bacterial products in the cytosol [3, 4]. Activation of NLRs, like that of TLRs, by bacterial products can stimulate the nuclear transcription factor (NF)-*κ*B pathway, a key regulator of the proinflammatory response, activating genes that are involved in immune responses. NLRs can also activate caspase-1 which cleaves proIL-1*β* to active IL-1*β*. 

 Recent evidence has shown that a deficiency in NLR proteins associated with caspase-1 could alter IL-1*β* expression. Naip5 is a member of the NLR protein family, and A/J mice, which have a defect in the naip5 protein, exhibit a reduced ability to kill *Legionella pneumophila* compared to wild-type mice [5]. Previously, it was revealed that TLR2 recognizes the antigens from *Mycobacterium leprae *and is involved in the immune responses and intracellular signaling in macrophages [1, 2]. However, the association between *M. leprae* and NLRs in the immune response is not well defined. We therefore assessed the ability of *M. leprae* to induce caspase-1 activity and IL-1*β* secretion in macrophages from C57BL/6 and A/J mice, representing mouse strain with restrictive and permissive Naip5 alleles, respectively. 

## 2. Materials and Methods

### 2.1. Mycobacterium leprae

*M. leprae* was obtained from infected nude mouse footpads as described by Kang et al. [6]. Footpads from *M. leprae*-infected nude mice were dissected, soaked in 1% iodine solution, and chopped finely with no. 10 and 15 disposable scalpels. These samples were then homogenized in 2 mL of DPBS with 25–30 glass beads in a Mickle homogenizer (Mickle Laboratory Engineering Co., Surrey, UK). An aliquot of the supernatant was stained with Ziehl-Neelsen's stain for AFB, which was quantified by the procedure of Shepard and McRae [7].

### 2.2. Cell Lysate Preparation of M. leprae

*M. leprae *lysate (MLL) was used as a stimulant for activation of NLRs in murine macrophages. *M. leprae *lysate was isolated as previously described [6]. In brief, *M. leprae* from mouse footpads were suspended in sonication buffer (50 mM Tris-HCl, 10 mM MgCl2, sodium azide 0.02%, pH 7.4) and treated ultrasonically for 45 min at 75 W with a Sonifier 250 (Branson Ultrasonic, USA) in an ice-water bath. The sonicated material was centrigued at 12,000 × g for 30 min and supernatants were stored at −20°C as cell lysate.

### 2.3. Mouse and Macrophage

A/J and C57BL/6J mice were obtained from (Central Lab. Animal, Inc. Seoul, Korea). Murine peritoneal cells were obtained as described previously [8]. Primary peritoneal macrophages were obtained from mice 4 days after intraperitoneal inoculation of 3 mL of 3% thioglycolate. Peritoneal fluid was drawn through the abdominal wall with a 23-gauge needle. Fluid from mice was pooled and washed, total cell counts were determined using a hemocytometer, and the remaining fluid was centrifuged at 380 × g for 10 min at 4°C. Washed cell suspensions were adjusted to 10^6^ macrophages per ml in culture medium containing RPMI 1640 with 10% fetal bovine serum and antibiotics. Animal treatment and maintenance were carried out in accordance with the Principle of Laboratory Animal Care (NIH publication No. 85–23 revised 1985) and the Animal Care and Use Guidelines of Sahmyook University, Korea.

### 2.4. Macrophage Infection

Peritoneal macrophages were cultured and infected with *M. leprae* in a multiplicity-of-infection (MOI)-dependent manner. In some experiment the cells were treated with *M. leprae* lysates. Macrophages were also stimulated with LPS (derived from *E. coli* O111:B4, Sigma). Culture supernatants were assayed for mouse IL-12, TNF-*α*, and IL-1*β* by ELISA (DuoSet, R & D).

### 2.5. Caspase-1 Assay

Caspase-1 activity assays were performed *in vitro* as previously described [9] using the caspase-1 assay kit (Calbiochem). Cell lysates were centrifuged at 10,000 × g for 5 min at 4°C, and caspase-1 activity was measured. The total increase in the optical density at 405 nm versus that of the sample alone was then calculated. Caspase-1 activity was expressed as follows: (maximum OD_405_/microgram protein) × 10,000.

### 2.6. NF-*κ*B Activity

Cytosolic and nuclear extracts were isolated and assayed for NF-*κ*B activity by the colorimetric method (NF-*κ*B EZ-TFA Transcription Factor Assay, Upstate) according to the manufacturer's instructions.

### 2.7. Statistical Analysis

All data were expressed as mean ± SD. Student's *t* test was used to analyze the data for statistical significance (GraphPad Prism), and significance was accepted at *P* < .05.

## 3. Results

### 3.1. Caspase-1 Activity and IL-1*β* Secretion in Response to M. leprae Was Reduced in Macrophages from A/J Mice

**Figure 1:**
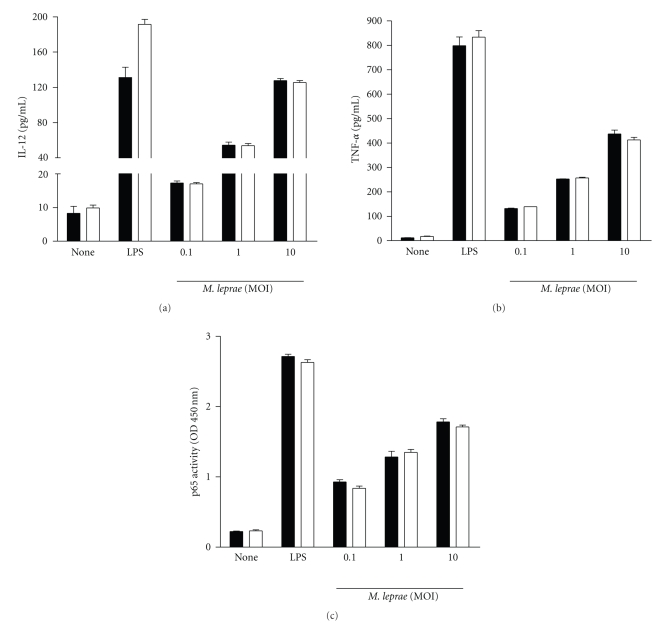
TNF-*α* and IL-12 production and NF-*κ*B activation in response to *M. leprae* infection in macrophages from A/J and C57BL/6 mice. Macrophages (10^6^) from C57BL/6 and A/J mice were treated with LPS (100 ng/ml) and *M. leprae* (MOI of 0.1, 1.0, and 10.0) for 18 h, and supernatants and cell extracts were assayed for cytokines (IL-12 and TNF-*α*) and NF-*κ*B, respectively. Open bar and closed bar represent macrophages from C57BL/6 and A/J mice, respectively. Data are representative of at least three independent experiments, each performed in triplicate.

Macrophages from C57BL/6 and A/J mice were infected with *M. leprae* and the levels of IL-12 and TNF-*α* produced by macrophages were measured by ELISA. The production of two cytokines was similar in macrophages from both mice (Figures 1(a) and 1(b)). NF-*κ*B activity levels measured with the p65 activity kit were also similar in both macrophages following infection with *M. leprae* (Figure 1(c)).

**Figure 2:**
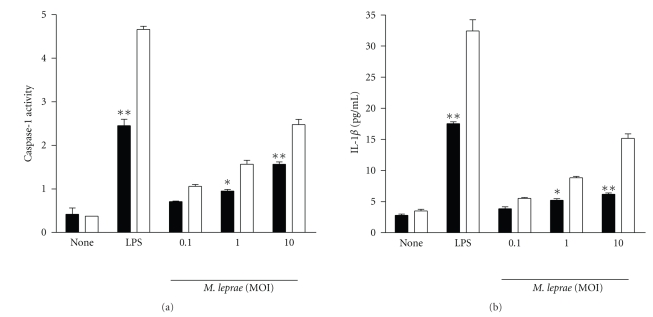
Caspase-1 activity and IL-1*β* production in response to *M. leprae* infection in macrophages from A/J and C57BL/6 mice. Macrophages (10^6^) from C57BL/6 and A/J mice were treated with LPS (100 ng/ml) and *M. leprae* (MOI of 0.1, 1.0, and 10.0) for 18 h, and supernatants and cell lysates were assayed for IL-1*β* concentrations and for caspase-1 activity, respectively. Open bar and closed bar represent macrophages from C57BL/6 and A/J mice, respectively. Data are representative of at least three independent experiments, each performed in triplicate. *P < .05; **P < .01.

 A previous report showed the differences in *Legionella*-induced IL-1*β* levels between A/J and C57BL/6 macrophages [5]. We measured the activation of caspase-1 after infection of macrophages with *M. leprae *because the maturation and secretion of IL-1*β* is dependent on the activation of caspase-1. Caspase-1 activity was lower in macrophages from A/J mice than in those from C57BL/6 mice (Figure 2(a)). We next studied the production of IL-1*β* during infection with *M. leprae*. As expected, IL-1*β* production was rather low in A/J macrophages whereas significant levels of mature IL-1*β* were measured in the culture supernatants of C57BL/6 macrophages (Figure 2(b)). The reduction in caspase-1 activation and in IL-1*β* production was however only partial in cells from the A/J mice stimulated with either LPS or *M. leprae*.

### 3.2. Caspase-1 Activity and IL-1*β* Secretion by MLL Was Also Reduced in A/J Macrophages

**Figure 3:**
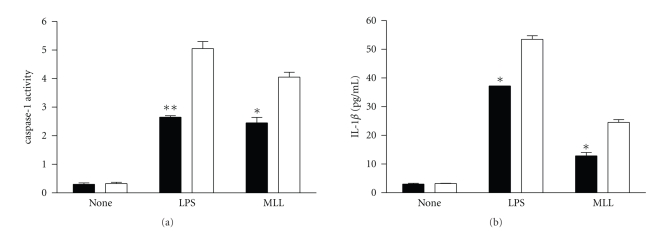
Caspase-1 activity and IL-1*β* production in response to *M. leprae* lysate (MLL) in macrophages from A/J and C57BL/6 mice. Macrophages (10^6^) from C57BL/6 and A/J mice were treated with LPS (100 ng/ml) and the cell lysates of *M. leprae* (1 *μ*g/ml) for 18 h, and supernatants and cell lysates were assayed for IL-1*β* concentrations and for caspase-1 activity, respectively. Open bar and closed bar represent macrophages from C57BL/6 and A/J mice, respectively. Data are representative of at least three independent experiments, each performed in triplicate. *P < .05; **P < .01.

Our previous data showed that upon exposure to MLL, macrophages produced the proinflammatory cytokine IL-12 and TNF-*α* [1, 2]. In addition, we also investigated caspase-1 activity and IL-1*β* production in response to *M. leprae* lysate (MLL) in macrophages from C57BL/6 and A/J mice. The results show that caspase-1 activity and IL-1*β* secretion were lower in macrophages from A/J mice than in those from C57BL/6 mice (Figure 3).

## 4. Discussion

NLR proteins play an important role in the surveillance of mammalian cytosol, providing a crucial interface between invading bacterial pathogens and the host immune system. Intracellular detection of the bacterium itself and of bacterially derived molecules might signal a danger to the host cell that is amplified and synergized with signals from cell surface receptors, such as the TLRs. The ultimate outcome of cytosolic NLR signaling is to trigger a proinflammatory response by activation and secretion of cytokines via the NF-*κ*B pathway and the inflammasome [10, 11].

 IL-1*β* is one of proinflammatory cytokines and is expressed following an inflammatory stimulus. In the inflammasome, which induces caspase-1-mediated generation of IL-1*β*, IL-1*β* has a critical role in the prevention of intracellular pathogens, including *Shigella, Salmonella, Listeria, Legionella, Francisella*, as well as *Bacillus anthracis *[10, 12, 13].

 Our data suggest that allelic difference in Naip5 found between A/J and C57BL/6 mice [5] partially controls caspase-1 activity and IL-1*β* secretion by macrophages in response to *M. leprae* infection. 

 Previous studies concluded that infection with *Legionella* activates Naip5 by delivering flagellin through its type IV secretion system, which then induces Ipaf-mediated caspase-1 activation and cell death to restrict *Legionella* replication [5, 14–16]. *M. leprae* is an intracellular pathogen and may also translocate into the cytosol [17] in which is able to interact with some NLRs. We also used MLL as another stimulant, and the results were similar to the data shown in *M. leprae* strain. Although flagellin is responsible for naip5-mediated caspase-1 activation in response to legionella, we suggest that presumably another component of *M. leprae* is responsible for its interaction with Naip5. Future study should include the analysis of cell wall components of mycobacteria and how they correlate with immune modulation via Naip 5.

 The previously described susceptibility of Legionella was shown at low MOIs of 1 or 0.5 [5]. Our study used at MOIs 0.1, 1.0, and 10 of *M. leprae* per macrophage and the differences in response to *M. leprae* in macrophage from the two mouse strains *were not* significant unless MOIs of 1 or 10 were used. Because *M. leprae* is slow-growing bacteria (doubling time is about 21 days) in contrast to rapid-growing *E. coli* and legionella, it is likely that the response of macrophages to *M. leprae* is induced at high MOI. 

 In our future studies, we will investigate whether susceptibility to mycobacterial infection, such as tuberculosis and leprosy, is increased in the absence of caspase-1 or IL-1*β*, as would indicate the importance of the inflammasome in host defense against mycobacterial infection. In addition, a downstream molecular target of Naip5 will be identified.

## 5. Conclusions

Although we did not find a component of *M. leprae*, which is responsible for the interaction with Naip5, it is likely that Naip5 is partially required for caspase-1 activation and IL-1*β* secretion by macrophages in response to *M. leprae* infection in our study using A/J mice, suggesting the possibility that NLRs may have a role in the innate immune response to *M. leprae*.
